# Thoughts and exploration of spatial planning system in the new era

**DOI:** 10.1007/s44243-022-00005-4

**Published:** 2023-01-10

**Authors:** Kai Wang, Jingjing Hu

**Affiliations:** China Academy of Urban Planning & Design, Beijing, China

**Keywords:** Spatial planning, Planning practice, Planning system, Technical routes

## Abstract

Spatial planning is a core requirement and a major initiative in the construction of ecological civilization in China, and the related work is in a process of gradual improvement and perfection. Based on the need of natural environment, urbanization and human well-being, this paper discusses the contents and technical directions of the current spatial planning in China from the national, regional and habitat levels, in the hope that it will be benefit current planning practice and provide references for further refinement of spatial planning system and technical routes in China.

## Ecological civilization and China’s spatial planning system

### The spatial planning system is a concrete practice in materializing the idea of ecological civilization in the new era

The idea of ecological civilization has been a major reform of China's philosophy and institution on development since the 18th CPC National Congress. The idea was proposed in China as early as 2007, and was elevated as the guiding principle for China's philosophy and action on development five years later. In recent years, with the proposal of the modernized governance system and the adoption of the Comprehensive Plan for the Institutional Reform of Ecological Civilization, ideas and practices concerning the fostering of ecological civilization, which has become the focus of China's future sustainable development, have been enriched.

It was pointed out at the fifth Plenary Session of the 19th CPC Central Committee that China's economy and people's demand for a better life has entered the stage of high-quality development, where the ecological environment has become significantly supportive. From the perspective of economic and industrial development, it has become the focus of the state to balance the relationship between production, people's life, and the ecological environment, to uphold the basic state policy of resource conservation and environmental protection, and to have that policy implemented in the spatial and industrial deployments. From the people-centered point of view, due to the new principal dispute between the ever-growing needs of the Chinese people for a better life and the reality of unbalanced and inadequate development, the Chinese people are currently concerned about the quality of the living environment, including the ecological environment. The development of ecological civilization covers the management of territorial spaces in many aspects.

### It is the central task of the spatial planning system to achieve high-level governance, high-quality development, and high quality of life with ecological development being placed at the top

It was proposed at the third Plenary Session of the 18th CPC Central Committee that the state would establish a spatial planning system. The Comprehensive Plan for the Institutional Reform of Ecological Civilization introduced in 2015 sees the establishment of the spatial planning system as one of the major tasks. Therefore, it is not only the starting point but also the central goal of the spatial planning system to put ecological development in the first place, based on which efforts will be made to fulfill the three core tasks of high-level governance, high-quality development, and high quality of life.

#### To achieve high-level governance, efforts should be made to ensure the coordinated development of "mountains, rivers, forests, fields, lakes and grasses" and the urban and rural areas

Under the current development mode, China's urbanization is facing such challenges as limited resources, polluted environment, and deteriorated ecosystem. In the National Urban System Planning (2006–2020) (Ministry of Housing and Urban–Rural Development, & China Academy of Urban Planning and Design, 2010), the China Academy of Urban Planning & Design ("CAUPD") calculates, after considering the slope, water, rainfall, etc., that around 20% of the 9.6 million square kilometers of territorial space can be inhabited by people, in which less than 9% of the land can be used for urban construction due to the existence of agricultural areas, forests, and wetlands. That is China's actual situation, and the fundamental reason why we need high-level governance (Wang & Wu, 2010).

Future development of territorial spaces must proceed with the basic understanding that "mountains, rivers, forests, fields, lakes, and grasses" and the spatial pattern of the urban and rural areas should form a shared community of life, work to improve the level of governance, respect nature, follow the way of nature, and protect nature. Only by doing that can we bring the genuine happiness to the people, build a truly beautiful China, and help the country stay competitive.

In its spatial planning of Nanchang, the CAUPD paid much attention to biodiversity. By analyzing and studying the interaction between wetland and the city, it proposed various planning strategies such as the hydro-fluctuation zone and the foraging ground, in order to protect the bird habitat and ensure the rational and sustainable utilization and management of the natural resources. When making plans for the Caohai Wetland in Weining, Guizhou province, which is a wintering area with the highest natural population density of the endangered black-necked cranes, the CAUPD studied and calculated specifically the construction of infrastructure from the perspective of the black necked cranes, so as to guarantee the coexistence of human and nature.

#### To promote high-quality development, efforts should be made to improve the efficiency and effectiveness of the utilization of territorial spaces

In the next 30 years, China will continue to promote urbanization at an appropriate rate. If the urban development continues to sprawl in an uncontrolled way, the consumption of energies and resources and the scale of construction will be unprecedented. Facing such challenges as the sharp conflict between the supply and demand of international energy and the multiplied pressure of global greenhouse gas emissions, General Secretary XI jinping pledged at the Climate Ambition Summit, which was held on December 12, 2020, that China will scale up its nationally determined contributions to address global climate change by peaking the carbon dioxide emissions before 2030 and achieving carbon neutrality before 2060; and that by 2030, China will lower its carbon dioxide emissions per unit of GDP by over 65 percent from the 2005 level, increase the share of nonfossil fuels in primary energy consumption to around 25 percent, increase the forest stock volume by 6 billion cubic meters from the 2005 level, and bring its total installed capacity of wind and solar power to over 1.2 billion kilowatts. These figures suggest that China has been more resolute and powerful than ever to promote the comprehensive green transformation of economic and social development in the process of promoting high-quality development, and territorial and urban spaces constitute the most important battlefield for China's green development of energy conservation and emission reduction (Wang & Chen, 2013).

However, at the present stage, the extensive urban spatial development mode and the inefficient use of construction land have led to the unbalanced allocation of territorial spatial resources and greatly aggravated the pressure of resources, the environment, and the ecosystem. Take the construction of new urban areas as an example. According to the data of the CAUPD, by 2018, there were more than 3,800 new urban areas in China, including 19 state-level new districts, 552 state-level development Zones, 1,991 provincial development zones, and 1,284 new urban areas below the provincial level. The average completion rate of these new urban areas is 55%. Separately calculated, the average completion rate of the state-level new districts, the state-level development zones, the provincial development zones, and the functional new townships is 55.5%, 47%, 39.5%, and 29.7%, respectively. It is of great urgency to improve the efficiency and effectiveness of the utilization of territorial spaces (Wang et al., 2020).

#### To lead a high-quality life, efforts should be made to improve the supply quality of territorial spaces

Since the 19th National Congress, China has found itself at a new development stage. The principal challenge facing the Chinese society is the gap between unbalanced and inadequate development and the people's growing expectation for a better life, and its urbanization also shifts from rapid growth to high quality development, i.e., from the traditional urbanization of land and things to the new people-centered urbanization, in which urbanization is promoted by meeting the needs of people. The primary task in supplying the territorial spaces is to create a high-quality living environment to attract qualified personnel and gather people, and establish an urban public service system to help the migrant agricultural population become urban citizens.

Across the world, countries and cities with a per capita GDP of more than $10,000 have seen a shift from the pursuit of economic growth to that of human happiness. It has become a shared commitment of the world's major cities to work for people's happiness, create a livable environment, and promote sustainable development. Some global cities, though not big in terms of economic volume, are famous for their living environment and life quality. Environment and landscape, building of public space, convenient travel, cultural atmosphere, housing and services, and community construction are things that concern those cities the most.

Through their projects in Nanjing, Hangzhou, and Chengdu, the CAUPD finds that the quality of life and the unique charm of the city are critical to attract qualified personnel, and many regions and cities will strive for characterized and differentiated development with efforts made in such areas as culture, creativity, tourism, elderly care, business, logistics, and organic farming. During that process, ecological green space, innovation ability, cultural development, life quality, and resilience and security will become the key competitiveness in the new wave of urban development.

## Key points of China’s spatial planning system in the new era

Territorial spaces have multiple attributes, of which economic, social, and ecological attributes are the most important ones. The past three decades have seen a significant change in the direction and approach of China's governance, which is represented by the shift in the country’s territorial spaces from economic growth first, to equal emphasis on economy, society, and environment, and finally to ecological development first, with social and economic development included, in the new era.

At present, China's spatial planning system has been initially established at the national, provincial, municipal, county, and town (township) levels, and is divided into three categories: master plan, detailed plan, and special plan. Yet the whole system is still in the process of reconstruction and requires continuous improvement, and more discussions are needed for the concrete work and working methods. Based on the planning practice of the CAUPD in the past decade, the author gives some opinions below (Wang, 2014).

### We should integrate the development of resources and environment nationwide with urbanization, and highlight the role of national spatial planning in guiding national urbanization policies

National spatial planning is the top-level design of the spatial planning system and plays an irreplaceable role in the country's urbanization policy. One thing that the CAUPD has learned from the two studies of the national urban system planning is that the national spatial planning is a complex and systematic project which involves multiple departments and fields, and that as the most important approach for countries to achieve the coordinated development between economy, society, environment, and region, it is the core function of the central government and the bellwether of the national urban and rural planning.

In a nutshell, the core content of the two editions of the national urban system planning published by the CAUPD is about how to help China stay competitive in sustainable development, specifically, in the five areas of nature, people, society, inhabitation, and support network, based on the resources and environment.

In the study of the national urban system planning (2005), the CAUPD makes the following suggestions: specifying the protection and management of precious resources at different levels based on the sustainable development of cities and towns; identifying internationally competitive cities and areas in the context of economic globalization; identifying key areas of urbanization through scientific forecast of population growth and flow; establishing a new regional coordination mechanism based on the regional collaborative development; building a dynamic multiple urban space structure based on the uncertainty of development; promoting changes in the way of urban economic growth under the guidance of the new industrialization; establishing a planning system integrating the three categories of plans based on the effective management of space; and creating a think-tank for urban spatial planning to make sound decisions on planning. The main idea is to integrate the development of resources and environment nationwide with urbanization, and to promote the strategic guidance of China's urbanization policy and make reasonable use of territorial space resources through intensified efforts in the protection of resources, the promotion of development, space governance, and institutional innovation.

In the national urban system planning study in 2015, the CAUPD did its work in the six aspects.

1. It built a comprehensive and open urban spatial pattern, emphasizing openness, networking, and multi-centers, from the interior to the exterior, from the east to the west, the coordination of land and sea, and the consolidation of the border areas.
2. It formulated a regional strategy featuring strategic planning of the west, rejuvenation of the northeast, integration of the coastal areas, and connectivity of the east, the middle, and the west.
3. It identified the differentiated urban spatial pattern by classifying four types of regions according to the suitability of people's living conditions and the carrying capacity of resources and the environment.
4. It made strategic judgments at different levels, and within different regions and groups to answer the question of "where do people come from and where do they go".
5. It developed the urbanization strategies based on the people and local conditions, with emphasis being placed on the strategies concerning three types of migrant population, i.e., "orderly promoting the urbanization of the migrant agricultural population across provinces, guaranteeing the urbanization of the migrant agricultural population outside the county and within the province in accordance with the principle of proximity, and supporting the urbanization of the migrant agricultural population in the county in accordance with the principle of proximity".
6. It stressed the idea of "rebalancing", with emphasis being equally placed on the competitive development of city clusters, and on the development of charming characteristic areas. It also initiated the "national charming scenic area".

The planning in 2015 inherited the idea and understanding of the planning in 2005, with its focus still on the integration of the development of the resources and environment nationwide with urbanization. Meanwhile, according to the new requirements made by the country for development, new explorations have been made and new ideas generated about the effect and value of resources and the environment, urbanization and population flow, and unbalanced regional development.

The urban system planning studies carried out in 2005 and 2015 have covered various stages of the development of territorial spaces and urbanization in China, yet the idea of integrating the development of resources and environment nationwide with urbanization has remained, and the effective strategic guiding role of the national spatial planning in the country's urbanization policy during different periods has been highlighted and gained much attention.

On the national spatial planning from the perspective of ecological civilization in the new era, we believe that we should stick to the idea of integrating the development of resources and environment nationwide with urbanization. While noticing the constraint of resources and the ecological environment on the whole territorial spaces, and protecting the precious resources that can affect the national interests and help maintain the overall quality of people's living environment, we should also be aware of the reality that China is now dealing with domestic and international markets and resources, of the overall assessment of China's economic and urbanization progress, and of the necessity to prevent and correct the failures of the market economy. We can enhance our international competitiveness through spatial development.

### Multi-level regional spatial planning (at provincial and municipal levels, and across watersheds and regions, etc.) is the key to coordinate the relationship between resources, the environment, and development, and to realize cross-regional and cross-departmental collaboration

The logic lying beneath the construction of ecological civilization in the new era is the relationship between man and nature, in which the contradiction between the urban and rural development with man as the main body and the natural ecological environment is the key point. The main battlefield to solve this contradiction is the regional spaces, including provinces, cities, watersheds, and cross-regions. Ecological resources and environment should be protected in the whole regional space. In order to solve the limitations of urban development, we should have a systematic understanding of the regional spaces, correctly handle the contradiction between urban development and ecological environment, and give full play to the important role of the multi-level regional spatial planning.

At the regional space level, traditional urban and rural planning has played a significant role in promoting urbanization and urban construction and development, but it lacks implementation and management approaches at the regional level, has poor communication of mandatory content, is less rigid, and lacks effective supervision. Traditional land-use planning is effective at the regional level, but it does not give enough consideration to the diversified urbanization development and the strategic urban development.

Therefore, in the new era, the key in regional spatial planning is to stick to the bottom line, strengthen the multi-dimensional control of natural resources, and make reasonable use of the ecological resources; to see the differences between the regions and objects, and shift from the fixed and rigid "border control" to the flexible and effective "multiple controls". To be specific, regional space should be re-evaluated in accordance with the value of ecological civilization, and we should develop a proper understanding of the quantitative, shaped, demarcated, and sequenced management in the urban and rural development under different geographical conditions, with different characteristics of resources and environment, and in different development stages, so as to promote the formation of the efficient, balanced, and beautiful new territorial spaces.

In the collaborative planning of the Beijing-Tianjin-Hebei region, the CAUPD argued that it should first stick to the bottom line, analyzed the overall carrying capacity of regional resources and environment, and addressed such issues as the severe water condition, the poor ecological capacity, and the limited atmospheric capacity. At the same time, from the perspective of regional ecological security pattern, it delimited the urban development boundary step by step to guide the orderly and efficient expansion of urban space; controlled the scale of urban and rural land use in accordance with the principle of "controlling the increase, tapping the stock, and optimizing the structure". In this way, the coordination between resources and environment and the urban and rural development was effectively realized at the regional level, and the background foundation of urban construction and development was determined. Based on the overall carrying capacity of regional resources and environment, the CAUPD also proposed to establish a harmonious and livable modern urban system with integrated functions, so as to realize the coordinated and innovative development of cities and regions. With the goal of improving the quality of the regional space, its study focused on the innovation and demonstration of regional governance in three types of areas, i.e., prohibited urban development areas, areas with cross-border conflicts, and areas with policy support. In this way, the construction of ecological civilization ran through the whole process of coordinated development in the Beijing-Tianjin-Hebei region in terms of carrying capacity, competitiveness, and regulatory power (Wang, 2017).

### To promote development with ecological civilization, we must continue to attach great importance to the high-quality development of urban living environment

Scarce resources and a fragile ecological environment highlight the demand for the high-quality development of territorial spaces in China and for a high-quality living environment for urban and rural residents. WU Liangyong argues that the human living environment should be "centered on the coordination between man and nature, and take the human living environment as the research subject", and "take into full consideration external factors such as ecological environment, economic foundation, and cultural conditions". Globally, major developed countries generally regard a good living environment as the most important competitiveness of urban development and the most universal well-being of people (Wang & Wu, 2010).

The COVID-19 outbreak in early 2020 exposed the weaknesses and problems in China's current construction of the urban living environment in terms of health, safety and livelihood guarantee at the grassroots level. Therefore, it is inevitable that future urban planning and construction will be people-centered, observe the principles of greenness, safety, resilience, and health, and strive for a quality, diversified, and urban–rural integrated ecological and civilized living environment.

In its project of Haikou city, the CAUPD discarded the old belief that "development is expansion". Instead, bearing in mind the purpose of making urban planning for the people and the goal of building an international waterfront garden city, it insisted on intensive development, identified the total volume, limited the capacity, tapped the stock, added the quantity, and improved the quality. In terms of specific working methods, it improved the urban ecological environment based on respect for nature, compliance with nature, and protection of nature, and put forward systematic solutions centering on improving the sustainability and livability of the urban living environment quality to address the issues that were most concerned by the citizens.

In the master plan of the Chongming Island in Shanghai, the CAUPD proposed the strategies of "Plus Ecology" and "Ecology Plus". "Plus Ecology" is the core strategy for building Chongming into a world-class ecological island from the aspects of environmental quality, ecological space, water resources, biodiversity, climate, and energies. "Ecology Plus" is a strategy where an ecological way of life and production is achieved by improving, based on the three functional development orientations of agriculture, tourism, and innovation, the planning measures for the high-quality development of living environment, such as a livable environment, recreational facilities, and natural scenery. After all, lucid waters and lush mountains are invaluable assets (Wang, 2019).

In addition, in the national sponge city planning and pilot project from 2015 to 2019, the CAUPD was responsible for the planning technologies and consultations of the sponge cites. In its practices in the pilot cities such as Tianjin, Wuhan, Changde, Hebi, and Suining, the CAUPD managed to promote the construction of the urban water environment in a systematic way and improve the infrastructure in the urban ecological environment by addressing such issues as water safety, water pollution, water use, and the construction of water ecology. From the perspective of water, sponge city planning further explores the planning strategies and technical measures for the high-quality development of human living environment, enriching and expanding the connotation and extension of the spatial planning system.

## Conclusion

The construction of ecological civilization is a long-term plan for China's sustainable development in the future. Territorial spaces are the most important carrier of the construction of ecological civilization, and the establishment of the spatial planning system in the new era is a specific measure to implement the idea of ecological civilization. China's construction of spatial planning system is closely linked to the country's development and reform process to an extent that has never been seen before.

With the publication of documents such as the opinions and suggestions of the state on the spatial planning system and the supervision of its implementation, and the guidelines for making spatial plans at the provincial and municipal levels, continuous efforts are being made to promote the practice of spatial planning at the national, provincial, and municipal levels. Meanwhile, we should be aware that more work needs to be done to ensure that the technological route in the spatial planning should put ecological development in the first place and guarantee high-quality development.

This paper discusses and explores, based on the CAUPD's planning practices, the current spatial planning carried out in China, yet it is far from enough. How to make the planning constrained by a bottom line, how to adapt the planning to local conditions, how to make the planning satisfy the need of high-quality development, how to make people-centered planning, and how make the planning capable of dealing with international competitions and responding to the community of a share future for mankind, the answer lies in the extensive practice carried out by the numerous planning workers at different levels and in different fields, which is also the basis and prerequisite for China to keep improving the spatial planning system and better develop the national ecological civilization.

## Data Availability

Not applicable.
